# The influence of geographic ranges, climatic niches and temperature fluctuations on population variability

**DOI:** 10.1098/rspb.2025.0818

**Published:** 2025-07-23

**Authors:** Cleber Ten Caten, Lauren A. Holian, Tad A. Dallas

**Affiliations:** ^1^ Department of Biological Sciences, University of South Carolina, Columbia, SC, USA

**Keywords:** geographic range, population variability, temperature variability, geographic position, climatic niche, niche position

## Abstract

The existence of patterns in population dynamics across species geographic ranges and climatic niches is a pervasive idea in ecology. Population variability (i.e. temporal variability in population density) should hypothetically increase near range edges or niche limits because of less suitable environments in these areas, but the occurrence of such patterns remains largely unexplored. Further, fluctuations in temperature could pose demographic constraints on populations and also influence their variability. We used Breeding Bird Survey data to show that the population variability of 97 resident North American birds consistently increases towards their niche limits and in areas with more variable temperatures, but not towards their geographic range edges. However, our model has limited explanatory power, and phylogenetic history and species traits could not explain these results. These findings suggest that other factors, such as biotic interactions and resource availability, might be more important drivers of population variability in resident North American birds.

## 1. Introduction

A general goal in ecology is to understand how species densities change across geographic space and over time [1,2]. A commonly reported trend is that populations, the number of individuals that belong to the same species and occur in the same geographic location, are more abundant the closer they are to the species geographic range centre [3,4]. Species climatic niches (i.e. the range of environmental conditions that allow species to persist indefinitely) are a main factor limiting species geographic distributions [5,6], such that these environmental conditions are assumed to be closer to the species optimum in the centre of their geographic ranges, leading to higher density in these areas [3,7,8]. Despite being once regarded as a general rule [9], evidence for the occurrence of geographic patterns in population density is limited [7,8,10,11]. This lack of support for these geographic trends could be occurring because geographic edges do not reflect climatic niche limits for many species [12,13], leading to the suggestion that population dynamics are more related to the position of populations within species climatic niches rather than their position within species geographic ranges [14,15].

Perhaps a less explored consequence of patterns of population density across geographic and niche space is that population dynamics would be more variable the closer populations are to the edges of geographic ranges and climatic niches [7,8,16]. As these populations would have lower densities, demographic stochasticity becomes an important driver of their dynamics, leading populations to be more variable near range edges or niche limits [8,17,18]. Understanding how populations vary across species geographic ranges and climatic niches is of particular importance given that increasing fluctuations in population densities are associated with higher extinction risk [19,20], and the detection of such trends could inform conservation efforts [21]. However, differences in how population variability is structured across geographic ranges or climatic niches have rarely been assessed [7,8,18] despite being considered prevalent across species [22].

Different extrinsic (environmental) and intrinsic (demographic) factors may affect population variability irrespective of population position within the species climatic niche or geographic range. For example, increased fluctuations in environmental conditions lead to more variable population dynamics given that extreme environments are more likely to pose demographic constraints on populations [19,23]. Land-use change is currently a major driver of biodiversity change in terrestrial ecosystems that also affects population dynamics [24,25]. For example, habitat loss caused by land-use change can increase the mortality or lead to the displacement of individuals from a given area [26,27] and might also influence the variability of populations. Species traits, such as body size and dispersal ability, may also influence patterns of population variability across geographic and climatic space. For instance, fluctuations in population densities are often associated with changes in body sizes [28,29] while dispersal usually stabilizes population dynamics [30,31]. Several of these traits that affect population dynamics are phylogenetically conserved [32,33], indicating that there could be a phylogenetic signal in patterns of population variability [34].

**Figure 1.**
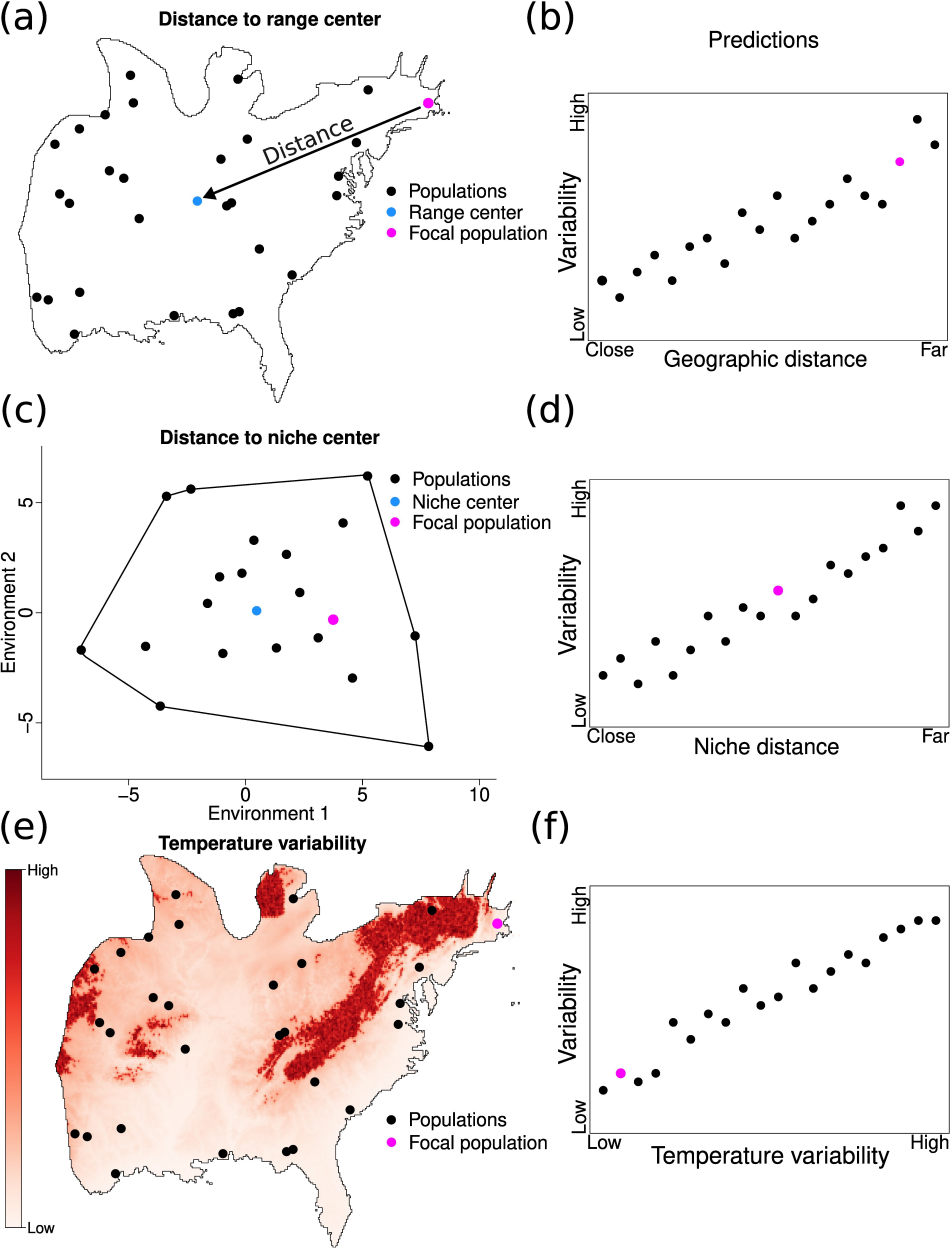
(a,b) Populations (black points) should vary more the farther they are from the centre of the species geographic ranges (blue point in (a)) because these locations have more unsuitable environments that would cause an increase in population variability. (c,d) However, range edges might not reflect niche limits, and populations found near geographic range edges might be close to the niche centre (blue point in (c)) of the species and be less variable than expected given their geographic position. (e,f). Temperature variability can further influence population variability, such that populations might be less variable when occurring in environments that do not fluctuate substantially. These factors can have different effects on the variability of a focal population (pink point), indicating that considering these three factors is key to understanding patterns of population variability.

The influence of geographic ranges and climatic niches on population variability represents a major knowledge gap in our understanding of population dynamics. Differences in environmental conditions that populations experience are considered important drivers of patterns of population dynamics across geographic ranges and climatic niches on population variability represents a major knowledge gap in our understanding of population dynamics. Differences in environmental conditions that populations experience are considered important drivers of patterns of population dynamics across geographic ranges and climatic niches [7,8,18], but the effects of environmental factors are often ignored during the assessment of these relationships. Here we address these gaps and examine the effects of climatic niche position, geographic range position and temperature variability on the population variability of 97 resident North American bird species (figure 1). Phylogenetic history and species differences in body size and dispersal ability were considered to assess their influence on these relationships. We hypothesized that population variability would be more strongly related to climatic niche position and temperature variability than geographic range position. We expected this given that climatic niches impose boundaries to the climatic conditions where populations can have positive growth rates and because temperature fluctuations can move populations along the species climatic niche, and in some cases, even beyond climatic niche limits, influencing their variability. Alternatively, geographic range edges might not represent climatic niche limits (electronic supplementary material, figure S1) and can have suitable environmental conditions that could buffer population variability [35]. We also expected that closely related species were going to exhibit similar patterns of population variability, and that species body size and dispersal ability would influence these relationships.

## 2. Methods

### (a) Estimating population variability

We used the North American Breeding Bird Survey (BBS) [36] data to estimate the yearly densities of 99 resident bird species. Species sampled by BBS were classified as resident based on [24]. The BBS is an annual standardized census of North American breeding birds. The surveys are conducted once per year by skilled volunteers during the breeding season where survey routes of ≈ 50 km are sampled every 1 km (i.e. a total of 50 stops) for about 3 min through point counts where all birds seen or heard within a 0.4 km radius are recorded. We only considered resident species because migratory birds might be exposed to different environmental conditions in their breeding and non-breeding ranges [37] that could affect their survival which would be challenging to consider in our analyses. Many migratory birds also have a substantial portion of their geographic ranges in areas outside of North America that are not sampled by BBS. Resident species could still have only a small portion of their geographic ranges sampled by BBS, but restricting our analysis to consider only resident species that had ≥70% of their geographic ranges sampled by BBS yielded the same results as when considering all resident species (see electronic supplementary material for details). BBS data were obtained through the bbsAssistant R package [38].

We used the data sampled between 1997 and 2019 to estimate population variability for the species considered in our study. The species considered in our study have been recorded in a total of 4803 routes since 1997. Each route has been sampled on average for 15 ± 7 years and an average of 305 ± 1127 routes were surveyed by BBS yearly between 1997 and 2019 (electronic supplementary material, figure S2). Population variability was estimated as the coefficient of variation (hereafter CV; CV=σ/μ), relating the standard deviation in temporal dynamics of a population (σ) to its mean (μ) in density in a site. Only sites where species were recorded for at least five different time periods (mean of 37 ± 1600 sites across species) were considered in our calculations. Population variability was estimated considering only the years a species was observed to occur in a target site. We also explored whether a more conservative estimate of population variability, only considering sites where species were observed to occur in at least 10 years, influenced the observed relationships. Alternatively, we also considered a less conservative estimate of population variability, where we assigned a density of zero to sampling events where a species was not recorded in a site, but that sampling event was done between the first and last year that a species was recorded at a given site to investigate whether this assumption affects our results.

### (b) Estimating geographic ranges, realized climatic niches and temperature variability

We estimated species geographic ranges as minimum convex polygons considering all BBS sampled points. The minimum convex polygon is defined as the smallest area that connects all sampled points that a species was recorded at with no interior edge. We opted to build our own polygons rather than using expert-derived ranges because of potential problems associated with using expert ranges. For example, expert estimates of a species geographic range can be disjunct, which can lead to the centre of the range falling outside the species geographic range and there might also be observations that fall outside of expert ranges [39], which could affect the assessment of these relationships. The centre of the species geographic range was defined as the centroid of the species ranges. Geographic range centres were also estimated using expert ranges obtained from BirdLife [40] rather than minimum convex polygons and we also used distance to range edge as an alternative measure of geographic range position and found similar results (see electronic supplementary material).

To estimate the realized climatic niche of the species in our study, we used the 19 bioclimatic variables, consisting of quantities related to temperature and precipitation relevant to species life history (e.g. temperature seasonality, temperature of coldest month, etc.), available in the CHELSA database [41]. These variables are considered the main drivers of North American birds distribution and abundance [42–44]. Bioclimatic variables were obtained at a resolution of 0.008°, and were aggregated to 0.5° to match the resolution of sampled BBS sites. We performed a principal component analysis (PCA) over these variables, and used the first two axes (explained ≈ 80% of the variance in the data) to estimate realized climatic niches. We extracted environmental values from these two PCA axes and used minimum convex polygons to determine species realized climatic niches (figure 1). The centroid of these polygons was used to represent the centre of climatic species niches. Climatic niches were also estimated using expert ranges and we observed similar results (see electronic supplementary material for details).

To estimate temperature variability, we obtained monthly mean temperature data for the years of 1997−2019 also from the CHELSA database at a resolution of 0.008° that were aggregated to 0.5°. To account for the different number of cells potentially being aggregated across different latitudes due to conformal projection, we also reprojected and aggregated mean monthly temperature data using Behrmann equal area projection (see details in electronic supplementary material). We estimated the coefficient of variation in temperature for each year and site where species were sampled to use as an estimate of temperature variability that populations experienced.

### (c) The relationship between population variability and geographic ranges, climatic niches, and temperature fluctuations

To evaluate how population variability was related to geographic range position, climatic niche position and temperature variability, we fit linear mixed models. Two species (*Strix nebulosa* and *Megascops kennicottii*) that were sampled in fewer than five sites for at least 5 years were removed from our analyses. Population variability was the response variable and geographic range position, climatic niche position and mean annual temperature variability were fixed effects in these models. Species were used as random effects (considering a reduced-rank covariance structure) to estimate the slope of the relationship between population variability and the fixed effects for each species separately. Thus, our models had random slopes, but not random intercepts, as random effects. All variables were standardized to have a mean of zero and a standard deviation of one prior to fitting the models. Linear mixed models were fit using a Gaussian family with the glmmTMB R package [45] and we performed model diagnostics (see electronic supplementary material, figure S3 for details) and estimated model goodness of fit (Nakagawa’s conditional $R^{2}$) using the performance R package [46]. This conditional $R^{2}$ takes fixed and random effects into account when estimating the model goodness of fit.

### (d) Assessing the effects of phylogeny and species traits on patterns of population variability

Information on body size and dispersal ability for each species was obtained from the AVONET database [47]. We used body mass as a measure of body size and the hand-wing index as a measure of dispersal ability. Measuring the dispersal ability of birds is a challenging task, and for that reason, biometric indices have often been used to describe their dispersal ability [48]. The hand-wing index considers information on flight efficiency and metabolic demands [49,50] and is positively related to the dispersal ability of birds [51]. We used the crosswalk taxonomy from AVONET [47] to link the taxonomies from BirdLife and BirdTree [52] to obtain phylogenetic information for the 97 resident species considered in our analyses. We removed *Aphelocoma woodhouseii* from our phylogenetic analyses because its equivalent species in BirdTree was a duplicate in our dataset (i.e. it was already present in our dataset). Thus, the phylogeny we used to assess whether species traits affect trends in population variability had 96 species.

We extracted the random slopes for each species from our linear mixed model and estimated Pagel’s λ and Blomberg’s K to assess whether there is a phylogenetic signal in how population variability is structured as a function of geographic range position, climatic niche position and temperature variability across species. Phylogenetic signal was estimated using the phytools package [53]. We also fit three phylogenetic generalized least squares (PGLS), one PGLS for each predictor considered in the linear mixed model, to explore how species slopes changed as a function of log-transformed body size and log-transformed dispersal ability. PGLS models were fit using the caper R package [54].

## 3. Results

### (a) Is population variability related to geographic range position, climatic niche position or temperature variability?

We found that the population variability of resident North American birds increases near climatic niche limits and in areas that have more variable temperatures (table 1). These results were consistent regardless of how population variability, climatic niches and temperature variability were estimated (see electronic supplementary material). The relationship between population variability and geographic range position was inconsistent and depended on how population variability was estimated, but not on how geographic ranges or range position were estimated (electronic supplementary material, tables S1 and S2). There was no relationship between population variability and geographic range position when population variability was estimated considering cases where species were observed at least 5 years in a site. However, these relationships became significant when zeros were considered when estimating population variability or when we only used populations that occurred in sites for at least 10 years (electronic supplementary material, tables S3 and S4). Nevertheless, conditional $R^{2}$ in our models were often ≈ 0.01, suggesting that most of the variance in these relationships remained unexplained.

**Table 1.** Results from the linear mixed model show that population variability increases near climatic niche limits (niche) and in variable temperatures (temp.) but not near geographic range edges (range). However, this model had a conditional $R^{2}$= 0.01, suggesting that this model has low explanatory power. Bold *p*-values represent significant relationships.

predictor	estimate	s.e.	*z* value	Pr (>|z |)
intercept	−0.001	0.005	−0.130	0.896
niche	0.043	0.012	3.485	**<0.001**
range	0.005	0.013	0.418	0.676
temp.	0.046	0.015	3.191	**0.001**

### (b) Do phylogenetic history and traits affect population variability responses across species?

**Figure 2.**
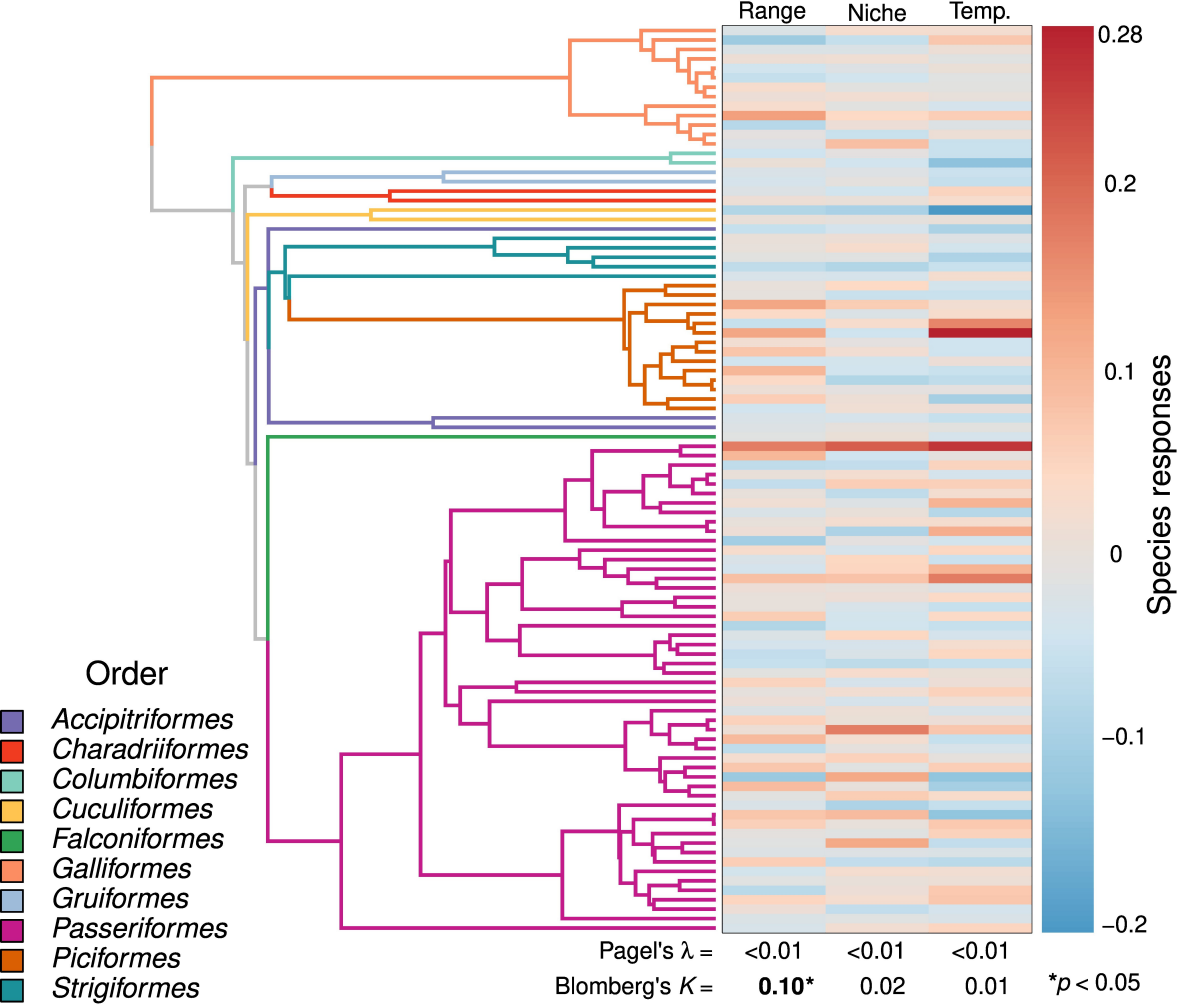
There is no phylogenetic signal (Pagel's λ and Blomberg's *K*) in the relationship (Species responses) between climatic niche position (Niche) and temperature variability (Temp.) and population variability across species. However, there was some evidence for phylogenetic signal (Blomberg's *K* = 0.10*, *p*‐value < 0.05) in the effects of geographic range position (Range) on population variability. In general, species traits could not explain these differences in how these predictors affected the population variability of different species.

There was no phylogenetic signal in the effects of climatic niche position (λ<0.01,p=1;K=0.02,p=0.79) or temperature variability (λ<0.01,p=1;K=0.01,p=0.92) on population variability across species. There was some evidence for phylogenetic signal (λ<0.01,p=1;K=0.10,p=0.03) in how population variability was related to geographic range position (figure 2). Species with smaller body sizes had more variable populations near geographic range edges, but not near climatic niche limits or in variable temperatures while dispersal ability was unrelated to patterns of population variability. These models were unable to explain most of the variance in patterns of population variability across species (adjusted $R^{2}\approx$ 0.01; table 2).

**Table 2.** Results from the phylogenetic generalized least squares show the effects of body size (body size) and dispersal ability (dispersal) on patterns of population variability across geographic ranges (range), climatic niches (niche) and variable temperatures (temp.). Note that even when significant relationships were observed (bold *p*‐value), these models still could not explain most of the variance in the data (i.e. small adjusted $R^{2}$ values).

condition	trait	estimate	s.e.	*t*-value	*p*-value	$R^{2}$
range	intercept	−0.022	0.039	−0.565	0.573	
	dispersal	0.022	0.015	1.482	0.142	0.04
	body size	−0.010	0.004	−2.419	**0.018**	
niche	intercept	0.037	0.038	0.970	0.334	
	dispersal	−0.006	0.014	−0.454	0.651	<0.01
	body size	−0.004	0.004	−0.954	0.343	
temp.	intercept	0.013	0.054	0.234	0.816	
	dispersal	0.006	0.020	0.311	0.756	<0.01
	body size	−0.007	0.006	−1.297	0.198	

## 4. Discussion

Population variability is predicted to increase in areas that have unsuitable environmental conditions such as near geographic range edges and climatic niche limits or in climatically variable locations [8,18,21], but there are limited empirical assessments of these expectations. We found that the population variability of resident North American birds consistently increases near climatic niche limits and in areas that have more variable temperatures, but not near geographic range edges. However, our models could not explain most of the variation in these relationships, and phylogenetic history and traits did not influence species differences in population variability. The limited explanatory power of these relationships may be because several factors, such as resource availability and biotic interactions, can simultaneously influence population variability [1,18], which could obscure these patterns across geographic and climatic niche spaces or in variable temperatures. The suitability of environmental conditions across geographic and climatic niche spaces might also not exhibit a homogeneous gradient of more to less suitable conditions from the centre towards the edges of ranges or climatic niches as previously suggested [3,14]. This would lead to noisier relationships between population variability and their position within geographic and climatic niche spaces of species.

Increased population variability near climatic niche limits and in variable temperatures is possibly due to the harsh boundaries that climatic niche limits impose on populations [4,55] and because temperature variability causes fluctuations in population growth rates and lead to more variable dynamics [56]. Geographic range edges may represent physical barriers, such as mountains and terrestrial boundaries, and not climatic niche limits for several species and can have suitable environmental conditions that might buffer population variability in these areas [12,13,35]. However, assigning a density of zero to sampling events where species were not recorded in sites or considering only sites that were sampled for over 10 years led to higher population variability near range edges. This suggests that sites that are only occupied temporarily by species influence observed patterns of population variability and that time series length might also be important to detect these relationships ([57]; see electronic supplementary material for details). Nonetheless, individual populations can exhibit adaptations to local environmental conditions, and have different climatic niche limits, that could mitigate their variability [12,58]. Such local adaptations, coupled with the occurrence of demographic compensation (i.e. opposing vital rate trends in different environments [59,60]), might weaken the influence of climatic niche position, geographic range position and temperature variability on population variability.

Species that have smaller body sizes often exhibit higher fluctuations in population density than larger-bodied species [61], but we did not observe a consistent effect of body size on patterns of population variability. The inability of body size to explain species differences in patterns of population variability can be due to other factors (e.g. level of ecological specialization and habitat loss) possibly regulating the effects of body size on population dynamics [62,63]. Although dispersal of individuals influences population dynamics [30,31], species dispersal ability was unrelated to population variability patterns. Experimental evidence shows that, in many scenarios, dispersal does not affect population variability [64], indicating that dispersal ability may be unable to explain species differences in patterns of population variability. The lack of phylogenetic signal in these relationships also suggests that the factors that influence patterns of population variability are highly variable across North American bird species.

The extent to which these findings are generalizable to other taxa is an interesting open question as the effects of geographic range position, climatic niche position and temperature fluctuations on population variability may depend on life history traits. Assessing the effects of habitat loss caused by land-use change on these patterns of population dynamics is also an important next step as species responses to such land-use changes may be mediated by their life history traits [24,25]. Considering the influence of species-level properties on the strength of these relationships may also further our understanding of these macroecological patterns. For instance, do species with larger geographic ranges also tend to have more variable populations near range edges? How does climatic niche breadth influence these relationships? By leveraging population dynamic theory [65,66], along with ideas from physiological ecology about species performance curves [18,67], it may be possible to generate testable predictions from theory about where populations would be most temporally variable. Incorporating the effects of density-dependent processes, such as competition, when evaluating population variability patterns may further elucidate these macroecological relationships.

## Data Availability

Data and R code to reproduce analyses are available on Figshare [68]. Supplementary material is available online [69].
